# Federal Nutrition Assistance Programs and UltraProcessed Food Intake among Preschool-Aged Children

**DOI:** 10.1016/j.cdnut.2025.107564

**Published:** 2025-09-27

**Authors:** Ashley Drengler, Evan C Sommer, Nadia M Sneed, Ellen McMahon, Kimberly P Truesdale, Donna Matheson, Tracy E Noerper, Lauren R Samuels, Shari L Barkin, William J Heerman

**Affiliations:** 1 Department of Internal Medicine, Vanderbilt University Medical Center, Nashville, TN, United States; 2 Department of Pediatrics, Vanderbilt University Medical Center, Nashville, TN, United States; 3 Center for Research Development and Scholarship, Vanderbilt University School of Nursing, Nashville, TN, United States; 4 Department of Nutrition, University of North Carolina, Chapel Hill, NC, United States; 5 Department of Pediatrics, Stanford University, Stanford, CA, United States; 6 Department of Nutrition, Lipscomb University, Nashville, TN, United States; 7 Department of Biostatistics, Vanderbilt University Medical Center, Nashville, TN, United States; 8 Department of Pediatrics, Emory University School of Medicine, Atlanta, GA, United States

**Keywords:** ultraprocessed foods, preschool children, low-income, federal nutrition programs, Supplemental Nutrition Assistance Program, Special Supplemental Nutrition Program for Women, Infants, Children

## Abstract

**Background:**

Among children in the United States, ultraprocessed foods (UPFs) account for ∼67% of daily calories, reflecting a low-quality diet. Among low-income preschool-aged children whose families participate in the Supplemental Nutrition Assistance Program (SNAP) or Special Supplemental Nutrition Program for Women, Infants, and Children (WIC), UPF consumption patterns have been understudied.

**Objectives:**

This study evaluated the association between SNAP and/or WIC enrollment and child UPF consumption and characterized the relationship between SNAP and WIC participation, food insecurity, and UPF intake among low-income, preschool-aged children.

**Methods:**

We conducted a secondary cohort analysis of an RCT for childhood obesity prevention that enrolled 610 predominantly Latino parent-child pairs from low-income families. The exposure was baseline participation in SNAP only, WIC only, both, or neither. The outcome was percentage of child caloric intake from UPFs. A linear mixed-effects model assessed the relationship between baseline assistance program participation and UPF intake over time, adjusting for sociodemographic covariates.

**Results:**

Among 582 eligible participants, median child age at baseline was 4.3 y (Q1, 3.6 y; Q3, 5.0 y); 91.4% (*n* = 532) of parents identified as Latino, 55.8% (*n* = 325) had household income <$25,000/y, and 42.8% (*n* = 249) had food insecurity. Approximately 21% (*n* = 124) of families used SNAP only, 12% (*n* = 68) of families used WIC only, and 54% (*n* = 316) used both. Median caloric intake of UPFs was 62.5% (Q1, 53.1%; Q3, 71.0%) at baseline. Neither assistance program use nor the interaction between assistance program use and household food insecurity was statistically significantly related to UPF intake.

**Conclusions:**

Among predominantly Latino preschoolers from low-income families, UPF intake is high (>60% of calories). The percentage of caloric intake from UPFs does not significantly differ by SNAP and/or WIC participation, regardless of food insecurity status.

This trial was registered at clinicaltrials.gov as NCT01316653.

## Introduction

In the United States, the federal government sponsors 2 major food safety-net programs, the Supplemental Nutrition Assistance Program (SNAP) and the Special Supplemental Nutrition Program for Women, Infants, and Children (WIC). These programs are designed to provide nutritional security, defined as having readily accessible and nutritious food, to individuals in low-income households. In 2020, ∼40 million Americans received SNAP benefits and 6.2 million received WIC benefits [1]. However, research has found that adults and children participating in these programs, when compared with income-eligible nonparticipants, often *1*) experience food insecurity [2], *2*) have a lower-quality diet [[3], [4], [5]], and *3*) consume more calories from highly palatable and habit-forming industrially processed food substances known as ultraprocessed foods (UPFs) (SNAP-only participants) [[6], [7], [8]].

Current dietary guidelines recommend a nutrient-dense dietary pattern rich in whole foods (eg, fruits, vegetables, low-fat dairy, and nuts/seeds) that minimizes highly processed foods containing added sugars, saturated fat, and sodium for health promotion across the lifespan [9,10]. A high-quality, nutritious diet is associated with improved health outcomes in pediatric populations [11,12]. This shift toward a whole food paradigm offers little room for the consumption of UPFs, which often contain limited or no whole food ingredients [8,[13], [14], [15]]. Policymakers have recently expressed concerns related to the effects of UPF intake on human health, and a number of states have introduced bills that would restrict or limit access to additives and certain food products (eg, processed meats) commonly described as UPFs [16]. Recent studies have demonstrated associations between UPF consumption and multiple chronic diseases, including obesity, cardiovascular disease, hypertension, cancer, and cerebrovascular disease [[17], [18], [19]]. Of concern is that UPFs have also been linked to chronic health conditions such as metabolic syndrome [20] and obesity among children [21,22].

In recent decades, UPF consumption has steadily increased among United States youth aged 2–19 y. From 1999 to 2018, total energy intake from UPFs increased from 61% to 67% [23], a trend that coincides with persistently low diet quality scores in this age group [24]. In addition, children from low-income households and non-Hispanic Black and Mexican American youth have disproportionately higher rates of UPF consumption than children from middle-income households or children who identify as non-Hispanic White [23]. A recent study analyzing NHANES data from over 9000 representative adults found that food insecurity was associated with increased consumption of UPFs [7]. Yet, the degree to which children whose families receive SNAP and/or WIC benefits consume UPFs has been understudied, and there are very little data about UPF consumption among preschool-aged children. Given that food insecurity is associated with a lower diet quality and is more prevalent among low-income and minority families with children [[25], [26], [27], [28], [29]], studies are needed that characterize the relationship between food insecurity, SNAP and WIC participation, and UPF intake, particularly in low-income preschool-aged children.

The objective of this study was to evaluate the extent to which receiving SNAP and/or WIC benefits was associated with consumption of UPF in preschool-aged children from low-income families. We also sought to test whether food insecurity moderated those associations, considering that over half of daily energy requirements for low-income children are consumed exclusively from UPFs [30]. Such findings could inform future changes in diet quality within SNAP and WIC programs to support the nutritional security of millions of low-income families.

## Methods

### Study design

This study was a secondary analysis of data from a previously conducted RCT. The Growing Right Onto Wellness (GROW) trial was an RCT intended to prevent childhood obesity via a 3-y family-centered healthy lifestyle intervention [31]. Parent-child pairs with children aged 3–5 y were enrolled from underserved neighborhoods in Nashville, TN. Parent-child pairs were recruited from 54 physicians’ offices and community settings. Families were consented and enrolled from August 2012 to May 2014. Data were collected at baseline and 12, 24, and 36 mo; the 36-mo follow-up was conducted between October 2015 and June 2017. The trial did not achieve its primary end point of preventing child obesity but did result in fewer calories consumed over time among children in the intervention compared with those consumed in those in the control group [32]. The original GROW trial adhered to all ethical standards outlined by the Helsinki Declaration. The Vanderbilt University Medical Center institutional review board and a National Heart, Lung, and Blood Institute–appointed data and safety monitoring board approved the study protocol and conducted routine evaluations of participant safety and protocol adherence throughout the trial. Informed consent was obtained by bilingual data collectors in participants’ language of choice.

### Setting/participants

The principal sites for the GROW trial were local community recreation centers in Davidson County, TN. In total, 610 parent-child pairs, mostly Latino, were enrolled. Important eligibility requirements included children who were 3–5 y of age at baseline, spoke English or Spanish, had a high normal to overweight BMI (≥50th and <95th percentile), and lived in Nashville and whose families qualified for ≥1 of the following federal assistance programs: Medicaid, TennCare, Free and Reduced Lunch, SNAP, or WIC.

### Exposure and outcome

The primary exposure was self-reported participation in a Federal Assistance Nutrition Program at baseline (reported as no program participation, SNAP only, WIC only, or both). The primary outcome was percentage of calories consumed from UPFs, collected at baseline and 12-, 24-, and 36-month time points. Child dietary intake was reported by parents using 24-h dietary recalls. At baseline, the 24-h diet recalls were collected within 30 days of the baseline survey where WIC and SNAP participation were reported. Recalls were collected using Nutrition Data System for Research software, and foods were categorized based on the extent and purpose of processing according to the Nova classification system [8,33,34]. Although the goal was to collect 3 recalls, 1–2 weekdays and 1 weekend day, per measurement occasion, data were analyzed for participants who had ≥1 eligible diet recall. We calculated the total daily kilocalories for each recall day and excluded all days with total kilocalories of <350 or >3750 as likely errors, based on their potential biological implausibility among young children. This resulted in excluding 21 of 5567 total recall days (0.38%). We then calculated the percentage of calories consumed from UPFs across the eligible recalls for each child at each time point.

### Participant characteristics

We collected child baseline age, sex, height, and weight (used to calculate BMI *z*-score and BMI category) and the following family baseline characteristics: parent ethnicity, country of birth, education, household income, and food security status. Child height and weight were measured by trained data collectors at baseline. All measurements were taken twice with height measured to the nearest 0.1 cm and weight measured to the nearest 0.1 kg, with the mean between the 2 measurements used as the final value. In the event that 2 measurements were outside a predefined threshold (0.3 kg or 0.5 cm), a third measurement was collected with the mean of the 2 closest measurements used for the final value. All other baseline data were collected by parent report. Household food security was measured using the 6-item short form developed by the National Center for Health Statistics [35,36]. Two items on this scale have Likert responses that include never true, sometimes true, and often true. One item has Likert responses that include the following: almost every month; some months but not every month; and only 1 or 2 mo. Three items have yes/no responses. Based on scoring guidelines from the USDA [37], items were summed to create a raw score from which 2 categories were derived: food secure (raw scores, 0–1) and food insecure (raw scores, 2–6). Participants with partially missing food security data were categorized accordingly if the responses on any missing items could not have affected their categorization (eg, a participant with a score of 0 and 1 missing item could be accurately categorized as food secure, regardless of how they might have responded on the single missing item).

### Statistical methods

To evaluate whether reported use of a nutrition assistance program at baseline was associated with child consumption of UPFs over 36 mo of follow-up, we used a prespecified longitudinal mixed-effects linear regression model with 2 levels (time nested within child) and a random intercept parameter to allow individual variability. The primary outcome was the percentage of calories from UPFs at each of the 4 time points. Time was incorporated into the model using the discrete time point variable. Use of a nutrition assistance program at baseline [no program (reference), SNAP only, WIC only, or both] was the main analytic variable of interest. Baseline covariates in the model were selected a priori based on their hypothesized associations with both the exposure and outcome (ie, potential confounders) and included the following: child age (years), sex (male compared with female), baseline BMI *z*-score, parent education (high school incomplete compared with high school complete or more), ethnicity (non-Hispanic compared with Hispanic), country of birth [United States (reference), Mexico, other Central or South American country, or other country], household food security (secure compared with insecure), and random assignment in the original RCT (control compared with intervention). The model included a 2-way interaction as follows:

$$\left(timepoint\right)\times \left({nutrition\;assistance\;program}_{baseline}\right)$$

It allowed estimates of the magnitude of the relationship between nutrition assistance program and percentage of calories from UPFs to vary over time. A prespecified secondary analysis was conducted to assess whether the relationship between nutrition assistance program participation and UPF consumption over time was moderated by food security status. The secondary analytic model was identical to the abovementioned model, except the 2-way interaction was replaced with a 3-way interaction as follows:

$$\left(timepoint\right)\times \left({nutrition\;assistance\;program}_{baseline}\right)\times \left({food\;security}_{baseline}\right)$$

along with all lower-order interaction terms. Because the 2- and 3-way interactions of interest include categorical variables with >2 categories, they were evaluated by Wald tests with 12 degrees of freedom to determine whether the constituent interaction parameters were jointly equal to zero. Additionally, because of the complexity of interpreting the regression coefficients in these models, the main results are presented graphically as model-based estimates of percentages of calories from UPFs at each time point with 95% CIs. The full coefficient results are included in Supplemental Table 1 and 2. Inclusion in the analytic sample required having data for all baseline covariates in the model and having outcome data for ≥1 time point. Missing outcome data were handled using a maximum likelihood procedure.

All analyses were conducted with Stata version 17.0 (StataCorp) [38]. Statistical significance was defined by a 2-sided *P* value of <0.05.

## Results

### Sample characteristics

Among 610 participants enrolled in the GROW trial, 582 (95.4%) had complete baseline covariate data and were included for analysis. Median (first quartile, third quartile) child age at baseline was 4.3 y (3.6, 5.0 y); 91.4% of parents identified as Latino, 55.8% had household income of <$25,000/y, and 42.8% reported household food insecurity. At baseline, 74 families (12.7%) were not enrolled in SNAP or WIC, 124 families (21.3%) were enrolled in SNAP only, 68 families (11.7%) were enrolled in WIC only, and 316 families (54.3%) were enrolled in both programs. Baseline demographics are shown overall and by WIC and/or SNAP participation in Table 1.

**TABLE 1:** Baseline participant characteristics by assistance program status.

Characteristic	SNAP only(*n* = 124)	WIC only(*n* = 68)	WIC and SNAP(*n* = 316)	No program(*n* = 74)	Total(*N* = 582)
Child					
Age (y)	4.8 (3.8, 5.4)	4.0 (3.5, 4.8)	4.2 (3.5, 4.8)	4.5 (3.6, 5.2)	4.3 (3.6, 5.0)
Sex					
Male	60 (48.4)	28 (41.2)	156 (49.4)	31 (41.9)	275 (47.3)
Female	64 (51.6)	40 (58.8)	160 (50.6)	43 (58.1)	307 (52.7)
BMI *z*-score	0.8 (0.5, 1.2)	1.0 (0.6, 1.4)	0.8 (0.4, 1.2)	0.8 (0.5, 1.1)	0.8 (0.4, 1.2)
BMI category					
Normal	83 (66.9)	37 (54.4)	211 (66.8)	49 (66.2)	380 (65.3)
Overweight	41 (33.1)	30 (44.1)	102 (32.3)	25 (33.8)	198 (34.0)
Obese	0 (0.0)	1 (1.5)	3 (0.9)	0 (0.0)	4 (0.7)
Family					
Parent ethnicity					
Non-Hispanic	13 (10.5)	4 (5.9)	22 (7.0)	11 (14.9)	50 (8.6)
Hispanic	111 (89.5)	64 (94.1)	294 (93.0)	63 (85.1)	532 (91.4)
Parent country of birth					
United States	16 (12.9)	6 (8.8)	28 (8.9)	13 (17.6)	63 (10.8)
Mexico	84 (67.7)	43 (63.2)	203 (64.2)	43 (58.1)	373 (64.1)
Other Central orSouth American country	22 (17.7)	19 (27.9)	79 (25.0)	17 (23.0)	137 (23.5)
Other	2 (1.6)	0 (0.0)	6 (1.9)	1 (1.4)	9 (1.5)
Parent education					
High school not complete	79 (63.7)	43 (63.2)	199 (63.0)	36 (48.6)	357 (61.3)
High school complete or more	45 (36.3)	25 (36.8)	117 (37.0)	38 (51.4)	225 (38.7)
Household annual income					
$14,999 or less	40 (32.3)	12 (17.6)	104 (32.9)	6 (8.1)	162 (27.8)
$15,000–$24,999	28 (22.6)	23 (33.8)	90 (28.5)	22 (29.7)	163 (28.0)
$25,000–$34,999	14 (11.3)	11 (16.2)	31 (9.8)	18 (24.3)	74 (12.7)
$35,000–$49,999	1 (0.8)	3 (4.4)	3 (0.9)	9 (12.2)	16 (2.7)
$50,000–$74,999	1 (0.8)	1 (1.5)	0 (0.0)	2 (2.7)	4 (0.7)
Do not know or no answer	40 (32.3)	18 (26.5)	88 (27.8)	17 (23.0)	163 (28.0)
Food security status					
Food secure	74 (59.7)	40 (58.8)	170 (53.8)	49 (66.2)	333 (57.2)
Food insecure	50 (40.3)	28 (41.2)	146 (46.2)	25 (33.8)	249 (42.8)
Random assignment					
Control	72 (58.1)	31 (45.6)	160 (50.6)	30 (40.5)	293 (50.3)
Intervention	52 (41.9)	37 (54.4)	156 (49.4)	44 (59.5)	289 (49.7)

Data are presented as median (first quartile, third quartile) for continuous variables and as *n* (%) for categorical variables.

Abbreviations: SNAP, Supplemental Nutrition Assistance Program; WIC, Special Supplemental Nutrition Program for Women, Infants, and Children.

Participants enrolled in SNAP and/or WIC descriptively had lower income, lower parental education, and a higher number of parents who were born outside of the United States than participants not enrolled in either program. Participants who were enrolled in WIC, SNAP, or both also had descriptively higher rates of food insecurity than participants not enrolled in either program. Baseline BMI was not meaningfully different between exposure groups.

### UPF consumption

**FIGURE 1:**
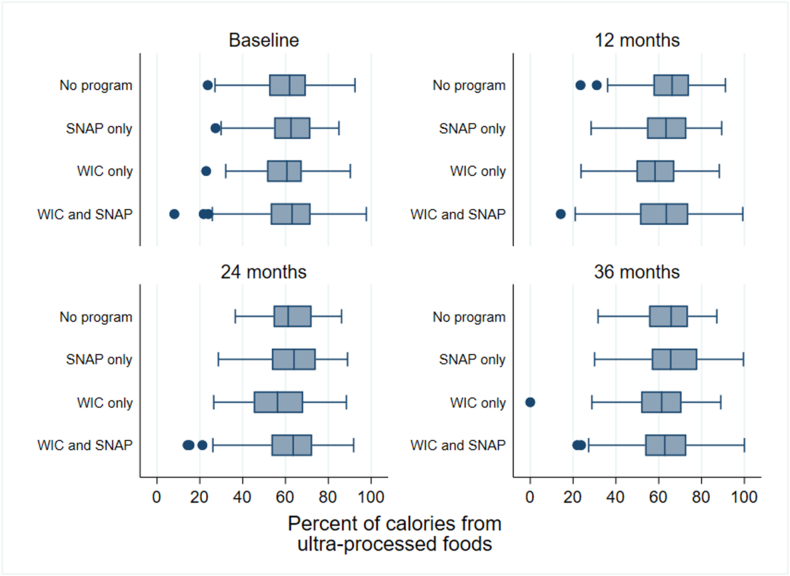
Percentage of calories from ultraprocessed foods by nutrition assistance program at each time point. Box plots indicate the observed distribution of the percentage of calories from ultraprocessed foods by nutrition assistance program at each time point. The line in the middle of each box represents the median. The line at the left and right of each box represents the 25th and 75th percentile, respectively. The left and right whiskers represent the lower and upper adjacent values, respectively. Dots represent outside values. SNAP, Supplemental Nutrition Assistance Program; WIC, Special Supplemental Nutrition Program for Women, Infants, and Children.

At baseline, the median (first quartile, third quartile) daily calories of UPF consumed was 690.6 (527.8, 911.6) raw kilocalories per day, corresponding to 62.5% (53.1%, 71.0%) of calories from UPFs. At baseline, the median (first quartile, third quartile) percentage of calories from UPF was 62.5% (54.7%, 71.6%) for children enrolled in SNAP only, 60.7% (51.4%, 67.6%) for children enrolled in WIC only, 63.0% (53.1%, 71.7%) for children enrolled in both SNAP and WIC, and 61.9% (52.3%, 69.5%) for children not enrolled in SNAP or WIC (Table 2). In general, median daily UPF consumption was between 100 and 200 cal/d lower for participants enrolled in WIC only than that for participants enrolled in the other groups at each time point. Box plots of UPF intake distributions at each time point by baseline SNAP and/or WIC participation are shown in Figure 1.

**TABLE 2:** Ultraprocessed foods and total caloric intake by time point and nutrition assistance program.

	SNAP only	WIC only	WIC and SNAP	Neither	Total
Baseline (*n* = 581)^1^					
UPF percentage	62.5 (54.7, 71.6)	60.7 (51.4, 67.6)	63.0 (53.1, 71.7)	61.9 (52.3, 69.5)	62.5 (53.1, 71.0)
Daily UPF kilocalories	729.6 (542.7, 935.8)	643.9 (492.0, 860.4)	694.4 (536.6, 923.2)	683.3 (515.7, 871.5)	690.6 (527.8, 911.6)
Daily total kilocalories	1216.7 (931.4, 1447.8)	1070.5 (861.3, 1379.0)	1142.3 (947.9, 1358.9)	1122.0 (911.3, 1391.5)	1151.8 (925.0, 1391.0)
12 mo (*n* = 461)					
UPF percentage	63.4 (54.6, 72.9)	58.3 (49.6, 67.3)	63.5 (51.2, 73.8)	66.2 (57.6, 74.1)	63.2 (52.9, 73.2)
Daily UPF kilocalories	773.4 (607.4, 913.0)	616.6 (510.7, 803.7)	736.7 (521.3, 971.1)	786.5 (623.9, 1021.8)	737.8 (546.7, 958.9)
Daily total kilocalories	1197.8 (1031.0, 1429.2)	1081.5 (923.2, 1271.0)	1192.4 (967.0, 1424.1)	1201.8 (1008.0, 1443.4)	1181.2 (970.6, 1420.1)
24 mo (*n* = 439)					
UPF percentage	64.0 (53.6, 74.1)	56.3 (45.2, 68.2)	63.6 (53.5, 72.4)	61.2 (54.4, 72.2)	62.4 (52.9, 72.3)
Daily UPF kilocalories	831.4 (606.7, 1065.5)	620.5 (476.8, 743.4)	742.5 (531.5, 992.8)	780.4 (557.9, 1025.9)	735.7 (540.7, 1006.2)
Daily total kilocalories	1316.9 (1049.6, 1561.1)	1070.6 (912.6, 1376.6)	1212.2 (939.2, 1439.4)	1228.0 (952.4, 1518.2)	1209.2 (953.1, 1487.3)
36 mo (*n* = 446)					
UPF percentage	65.6 (56.8, 78.0)	61.4 (51.8, 70.6)	62.8 (53.7, 72.9)	65.8 (55.5, 73.6)	64.3 (54.0, 73.6)
Daily UPF kilocalories	868.2 (632.2, 1166.6)	742.0 (553.7, 902.5)	765.9 (579.8, 1019.3)	835.9 (660.7, 1130.8)	785.8 (587.8, 1052.8)
Daily total kilocalories	1321.0 (1048.2, 1632.4)	1203.3 (1035.1, 1353.8)	1243.0 (1011.4, 1478.6)	1282.9 (1053.3, 1619.3)	1257.8 (1026.4, 1515.7)

Data are presented as median (first quartile, third quartile).

Abbreviations: SNAP, Supplemental Nutrition Assistance Program; UPF, ultraprocessed food; WIC, Special Supplemental Nutrition Program for Women, Infants, and Children.

1 The baseline sample size in this table is 1 less than the analytic sample (*n* = 582), because 1 participant was missing diet data at baseline but had data for ≥1 subsequent time point and was, therefore, included in the analysis.

### Association of WIC and/or SNAP participation with child UPF consumption

**FIGURE 2:**
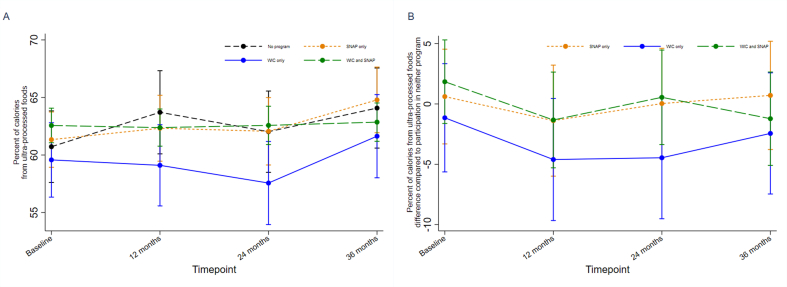
Model-estimated ultraprocessed food (UPF) consumption over time for each nutrition program: (A) estimated UPF consumption for each nutrition program; (B) estimated differences in UPF consumption for each nutrition program compared with participation in neither program. Graphical plots display estimates based on the primary longitudinal mixed-effects linear regression model evaluating whether nutrition assistance program use at baseline was associated with child consumption of UPFs over 36 mo of follow-up. Model adjusted for child sex, baseline age, and BMI *z*-score; parent education, ethnicity, and country of birth; household food insecurity; and random assignment in the original RCT.

**FIGURE 3:**
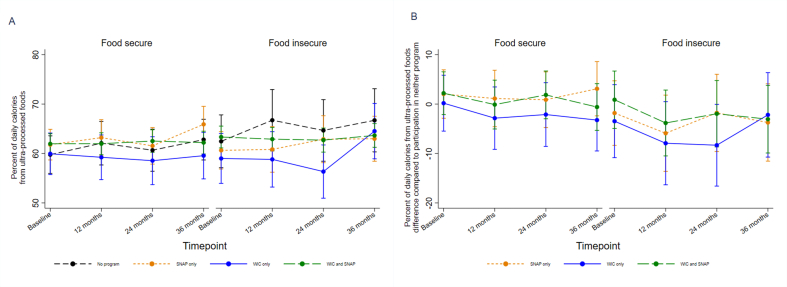
Model-estimated ultraprocessed food (UPF) consumption over time for each nutrition program by food security status. (A) Estimated UPF consumption for each nutrition program by food security status; (B) estimated differences in UPF consumption for each nutrition program compared with participation in neither program by food security status. Graphical plots display estimates based on the secondary longitudinal mixed-effects linear regression model evaluating whether the association between nutrition assistance program use at baseline and child consumption of UPFs over 36 mo of follow-up was moderated by food insecurity status. Model adjusted for child sex, baseline age, and BMI *z*-scores; parent education, ethnicity, and country of birth; household food insecurity; and random assignment in the original RCT.

The prespecified mixed-effects regression model did not detect a statistically significant association between SNAP and/or WIC participation and UPF consumption over time (Wald test: χ^2^ = 12.52; *P* = 0.4; df = 12; full model output in Supplemental Table 1). Model-based estimates of the percentage of calories from UPFs at each time point by baseline SNAP and/or WIC participation are shown graphically in Figure 2. The secondary regression model did not detect statistically significant moderation of the relationship between program participation and UPF consumption over time by food security status (Wald test: χ^2^ = 7.61; *P* = 0.8; df = 12; full model output in Supplemental Table 2). Figure 3 graphically displays model-based estimates of the percentage of calories from UPFs for the different programs at each time point by food security status.

## Discussion

In this cohort analysis of predominantly Latino children from low-income families, we did not detect a significant association between SNAP and/or WIC participation and child UPF consumption after adjusting for child and family sociodemographic characteristics. The modification of this association by household food security status was also not statistically significant. Regardless of participation in federal nutrition assistance programs, however, the median percentage of child caloric intake from UPFs was >60%. This finding highlights a potential opportunity for federal nutrition assistance programs to improve dietary quality. Interventions that focus on maximizing consumption of whole foods while limiting UPFs [39] could be a strategy to be considered in future research among low-income children and families.

The high percentage of UPFs consumed by children in the current study is consistent with prior research, which has reported that >60% of total calories consumed by United States children are from UPFs [23]. This is also similar to prior studies that reported that SNAP and WIC participants have a higher level of grocery store expenditures from UPFs [40,41]. The current analysis advances this previous work by focusing on intake, measured by 24-h diet recall data, instead of household purchasing practices, as the 24-h diet recall offers a more accurate representation of actual UPF consumption. The specific population of interest for the current study was predominantly Latino children from low-income families. This represents an important population subgroup who may have unique sociocultural needs that are best met by tailored interventions, which provides an opportunity for federal nutrition programs to improve overall population health. Consistent with previous studies, the rate of UPF consumption in this population of interest was high [23]. This may be attributed to the different diet-related disparities previously observed in certain population subgroups. For example, low-income households that identify as non-Hispanic Black or Latino often have reduced access to healthy foods [42] and experience disproportionately high rates of food insecurity [43,44]. In addition, previous research has shown that acculturation (ie, English permeation at home) is associated with higher levels of UPF consumption among Latino populations [45]. Further investigation is necessary to better elucidate the relationship between health disparities and UPF consumption, especially in the Latino population. However, using similar methods (ie, categorizing UPF consumption from 24-h diet recalls) has broad applicability for understanding the complex drivers of dietary quality and UPF consumption with public health implications for multiple populations.

Food insecurity, which has been shown to be more prevalent among minority and low-income families with children, is positively associated with consumption of lower-quality diets [[25], [26], [27], [28], [29]]. Because families with lower household income are more likely to be food insecure [44], we were interested in learning whether the association between SNAP and WIC participation and consumption of UPFs in children would differ by food security status. However, as noted earlier, the analysis did not detect a significant interaction. It is important to note that this may have been partially because the population enrolled in this study was exclusively from low-income households eligible for certain types of federal assistance. Although this criterion enabled the study to focus on an understudied population, it also likely reduced the number of participants who did not participate in a nutrition assistance program, a key comparison group, and precluded inclusion of a larger segment of the population who were not eligible for nutrition assistance. The small sample size of participants who were not enrolled in an assistance program (12.7%) reduced the ability to detect statistical significance in both models, especially in the context of 2- and 3-way interactions. Future studies that evaluate dietary quality among families from low-income households with food insecurity by participation in federal nutrition assistance programs compared with nonparticipation in federal nutrition assistance programs should be prioritized.

This study has several strengths, including its large sample size, the long-term and repeated follow-ups, and the wide range of covariates included. There are also several limitations in this study. Residual confounding and selection bias must be considered in this cohort study. Furthermore, the 24-h diet recall is subject to recall and social desirability bias. The baseline SNAP/WIC participation data are self-reported, and we have no data on how long or how often families used the programs after baseline. In addition, SNAP and WIC benefit levels vary by household size and income, which could affect purchasing power and food choices. However, our analyses did not account for differences in benefit amounts. Data collection occurred over multiple years but seasonal variations in food availability, cost, and consumption patterns, which could have affected UPF intake patterns differently across groups, were also not accounted for. Finally, the generalizability of this study is limited based on the selection criteria, including restricted BMI range, ethnicity, location, and the requirement that families be qualified for ≥1 relevant federal assistance program. Moreover, these data are from 2012 to 2017, which were before the COVID pandemic and population demographics and social conditions may have changed since then. Any potential baseline BMI differences that may exist between the groups in the general population may have been suppressed in this sample because of the restricted BMI range imposed by the eligibility criteria. Participants in this study were primarily Latino families living in the Nashville area; however, this is an important population to study due to the disproportionate burden of childhood obesity affecting Latino communities. Although the federal assistance eligibility criterion limited representation of participants who were not enrolled in a nutrition assistance program, it also enabled study of a unique group of low-income participants.

Among predominantly Latino children from low-income households, the percentage of calories from UPFs was not statistically significantly different by SNAP and/or WIC participation, regardless of food insecurity status. Regardless, the median percent of caloric intake from UPFs was high (>60%). Given the increasing prevalence of UPF consumption in preschool-aged children and their families, reducing UPF intake during early childhood represents an opportunity to improve diet quality to reduce diet-related health disparities.

## Author contributions

The authors’ responsibilities were as follows—AD, ECS, NMS, LRS, WJH: designed the research; ECS: performed the statistical analysis; AD, ECS, NMS, LRS, WJH: drafted the manuscript; EM, KPT, DM, TEN, SLB: contributed to the manuscript; WJH: had primary responsibility for the final content; and all authors: read and approved the final manuscript

## Data availability

Data described in the manuscript, code book, and analytic code will be made available upon reasonable request.

## Funding

This work was supported by funding from the National Heart, Lung, and Blood Institute (R03HL154243 and U01HL103620). NMS was supported in part by the following training grants T32HS026122 from Agency for Healthcare Research and Quality and 5KL2TR002245 from National Center for Advancing Translational Sciences. Data were collected and stored using REDCap, supported by grant UL1 TR000445 from the National Center for Advancing Translational Sciences at the National Institutes of Health. This content is solely the responsibility of the authors and does not necessarily represent the official views of the National Heart, Lung, and Blood Institute, the Agency for Healthcare Research and Quality, or Vanderbilt University.

## Conflict of interest

The authors declare no conflicts of interest.
