# Supplementary material for: Federal Nutrition Assistance Programs and UltraProcessed Food Intake among Preschool-Aged Children
Source: Curr Dev Nutr. 2025 Sep 27;9(11):107564. doi: 10.1016/j.cdnut.2025.107564 (PMC12581687; doi:10.1016/j.cdnut.2025.107564)
Supplement: Multimedia component 1 [file mmc1.docx]

**Supplementary Materials**

**Supplementary Table 1:** Primary mixed-effects regression output^1, 2^

|  | Coefficient | 95% confidence interval | *p-*value |
| --- | --- | --- | --- |
| Nutrition assistance program (ref: no program) |  |  |  |
| SNAP only | 0.6 | [-3.3, 4.5] | 0.760 |
| WIC only | -1.1 | [-5.6, 3.3] | 0.616 |
| WIC and SNAP | 1.8 | [-1.6, 5.3] | 0.297 |
| Timepoint (ref: baseline) |  |  |  |
| 12 months | 3.0 | [-1.1, 7.1] | 0.156 |
| 24 months | 1.3 | [-2.8, 5.4] | 0.529 |
| 36 months | 3.4 | [-0.7, 7.4] | 0.101 |
| Program X timepoint interaction (ref: no program X Baseline) |  |  |  |
| SNAP only X 12 months | -2.0 | [-7.3, 3.3] | 0.457 |
| SNAP only X 24 months | -0.6 | [-5.8, 4.7] | 0.828 |
| SNAP only X 36 months | 0.1 | [-5.1, 5.2] | 0.971 |
| WIC only X 12 months | -3.5 | [-9.3, 2.4] | 0.244 |
| WIC only X 24 months | -3.3 | [-9.1, 2.5] | 0.265 |
| WIC only X 36 months | -1.3 | [-7.1, 4.5] | 0.661 |
| WIC and SNAP X 12 months | -3.2 | [-7.7, 1.4] | 0.171 |
| WIC and SNAP X 24 months | -1.3 | [-5.8, 3.2] | 0.573 |
| WIC and SNAP X 36 months | -3.1 | [-7.5, 1.4] | 0.177 |
| Child baseline age | 0.9 | [-0.0, 1.8] | 0.057 |
| Child sex (ref: male) |  |  |  |
| Female | -2.3 | [-3.9, -0.7] | 0.005 |
| Child baseline BMI-Z | -0.5 | [-2.2, 1.3] | 0.604 |
| Parent education (ref: high school not complete) |  |  |  |
| High school complete or more | 0.2 | [-1.5, 1.9] | 0.804 |
| Parent ethnicity (ref: non-Hispanic) |  |  |  |
| Hispanic | -4.4 | [-9.6, 0.7] | 0.092 |
| Parent country of birth (ref: US) |  |  |  |
| Mexico | -5.5 | [-10.1, -1.0] | 0.017 |
| Other Central or South American country | -8.1 | [-12.8, -3.4] | 0.001 |
| Other | -6.2 | [-13.3, 1.0] | 0.091 |
| Food security status (ref: food secure) |  |  |  |
| Food insecure | 0.8 | [-0.9, 2.4] | 0.353 |
| Random assignment (ref: control) |  |  |  |
| Intervention | -2.4 | [-4.0, -0.8] | 0.003 |
| ^1^ Abbreviations: UPF: Ultra-processed foods; SNAP: Supplemental Nutrition Assistance Program; WIC: Special Supplemental Nutrition Program for Women, Infants, and Children (WIC).  ^2^ Primary longitudinal mixed-effects linear regression model that evaluated the association between nutrition assistance program use at baseline and child consumption of ultra-processed foods over 36 months of follow-up. Model adjusted for child sex, baseline age, and BMI Z; parent education, ethnicity, and country of birth; household food insecurity and random assignment in the original RCT. | | | |

**Supplementary Table 2:** Secondary mixed-effects regression output (moderation by food security status)^1, 2^

|  | Coefficient | 95% confidence interval | *p-*value |
| --- | --- | --- | --- |
| Nutrition assistance program (ref: no program) |  |  |  |
| SNAP only | 2.0 | [-2.9, 6.9] | 0.418 |
| WIC only | 0.2 | [-5.5, 5.8] | 0.950 |
| WIC and SNAP | 2.2 | [-2.1, 6.5] | 0.320 |
| Food security status (ref: food secure) |  |  |  |
| Food insecure | 2.7 | [-3.9, 9.3] | 0.420 |
| Program X food security interaction (ref: no program X food secure) |  |  |  |
| SNAP only X Food insecure | -3.8 | [-12.0, 4.3] | 0.355 |
| WIC only X Food insecure | -3.6 | [-13.0, 5.7] | 0.442 |
| WIC and SNAP X Food insecure | -1.3 | [-8.6, 5.9] | 0.721 |
| Timepoint (ref: baseline) |  |  |  |
| 12 months | 2.3 | [-2.7, 7.4] | 0.369 |
| 24 months | 0.9 | [-4.0, 5.8] | 0.719 |
| 36 months | 3.0 | [-1.8, 7.9] | 0.215 |
| Program X timepoint interaction (ref: no program X baseline) |  |  |  |
| SNAP only X 12 months | -0.9 | [-7.5, 5.6] | 0.787 |
| SNAP only X 24 months | -1.2 | [-7.6, 5.3] | 0.728 |
| SNAP only X 36 months | 1.1 | [-5.3, 7.4] | 0.740 |
| WIC only X 12 months | -3.0 | [-10.4, 4.3] | 0.417 |
| WIC only X 24 months | -2.3 | [-9.7, 5.1] | 0.544 |
| WIC only X 36 months | -3.4 | [-10.7, 3.9] | 0.356 |
| WIC and SNAP X 12 months | -2.3 | [-8.0, 3.4] | 0.428 |
| WIC and SNAP X 24 months | -0.3 | [-5.9, 5.3] | 0.905 |
| WIC and SNAP X 36 months | -2.8 | [-8.3, 2.7] | 0.319 |
| Food security status X timepoint interaction (ref: food secure X baseline) |  |  |  |
| Food insecure X 12 months | 2.0 | [-6.8, 10.7] | 0.661 |
| Food insecure X 24 months | 1.3 | [-7.3, 9.9] | 0.768 |
| Food insecure X 36 months | 1.2 | [-7.5, 9.9] | 0.784 |
| Program X food security status X timepoint interaction (ref: no program X food secure X baseline) |  |  |  |
| SNAP only X Food insecure X 12 months | -3.2 | [-14.1, 7.8] | 0.570 |
| SNAP only X Food insecure X 24 months | 1.2 | [-9.8, 12.2] | 0.831 |
| SNAP only X Food insecure X 36 months | -3.0 | [-13.9, 7.9] | 0.592 |
| WIC only X Food insecure X 12 months | -1.4 | [-13.5, 10.7] | 0.817 |
| WIC only X Food insecure X 24 months | -2.6 | [-14.6, 9.5] | 0.678 |
| WIC only X Food insecure X 36 months | 4.7 | [-7.4, 16.8] | 0.448 |
| WIC and SNAP X Food insecure X 12 months | -2.4 | [-11.9, 7.1] | 0.620 |
| WIC and SNAP X Food insecure X 24 months | -2.5 | [-12.0, 7.0] | 0.606 |
| WIC and SNAP X Food insecure X 36 months | -1.1 | [-10.6, 8.4] | 0.814 |
| Child baseline age | 0.9 | [-0.0, 1.8] | 0.054 |
| Child sex (ref: male) |  |  |  |
| Female | -2.2 | [-3.8, -0.6] | 0.007 |
| Child baseline BMI-Z | -0.4 | [-2.1, 1.3] | 0.617 |
| Parent education (ref: high school not complete) |  |  |  |
| High school complete or more | 0.1 | [-1.6, 1.8] | 0.866 |
| Parent ethnicity (ref: non-Hispanic) |  |  |  |
| Hispanic | -4.2 | [-9.4, 0.9] | 0.105 |
| Parent country of birth (ref: US) |  |  |  |
| Mexico | -5.5 | [-10.0, -0.9] | 0.018 |
| Other Central or South American country | -8.0 | [-12.7, -3.2] | 0.001 |
| Other | -6.1 | [-13.3, 1.1] | 0.095 |
| Random assignment (ref: control) |  |  |  |
| Intervention | -2.4 | [-4.0, -0.8] | 0.003 |
| ^1^ Abbreviations: UPF: Ultra-processed foods; SNAP: Supplemental Nutrition Assistance Program; WIC: Special Supplemental Nutrition Program for Women, Infants, and Children (WIC).  ^2^ Secondary longitudinal mixed-effects linear regression model that evaluated whether the association between nutrition assistance program use at baseline and child consumption of ultra-processed foods over 36 months of follow-up was moderated by food insecurity status. Model adjusted for child sex, baseline age, and BMI Z; parent education, ethnicity, and country of birth; household food insecurity and random assignment in the original RCT. | | | |
